# Age, Pain Intensity, Values-Discrepancy, and Mindfulness as Predictors for Mental Health and Cognitive Fusion: Hierarchical Regressions With Mediation Analysis

**DOI:** 10.3389/fpsyg.2019.00517

**Published:** 2019-03-07

**Authors:** Darren J. Edwards

**Affiliations:** Department of Public Health, Policy and Social Sciences, Swansea University, Swansea, United Kingdom

**Keywords:** pain, age, psychological flexibility, mental health, cognitive fusion

## Abstract

**Background:** Several studies have confirmed that higher levels of psychological flexibility predict better functioning for those suffering with chronic pain. However, few studies have investigated the role of the individual components of psychological flexibility within a chronic pain population in relation to aging specifically and the related indirect mediational processes.

**Aim:** The present study aimed to compare how age, pain intensity, mindfulness, and values-discrepancy predicted mental health and cognitive fusion separately. It also explored the indirect process relations through the use of a mediated analysis.

**Methods:** Two hundred and thirty three participants completed an online survey which included demographical questions as well as the following questionnaires; Short Form McGill Pain Questionnaire (SF-MPQ); General Health Questionnaire 12; Cognitive Fusion 7-Item Questionnaire (CFQ-7); Mindfulness Attention Awareness Scale (MAAS); and the Chronic Pain Values Inventory (CPVI). The relationships from the responses of the questionnaires and demographics were then analyzed through two hierarchical regression models followed by further mediation analysis.

**Results:** In the first model, values-discrepancy, pain intensity, and mindfulness all predicted mental health, but age did not. However, age did account for a significant portion of the variance in the second model when cognitive fusion was used as the dependent measure. It was also found that cognitive fusion mediated the relationship between age and mental health.

**Conclusion:** These results are discussed within the context of using indirect process relations of psychological flexibility and third wave therapies such as acceptance and commitment therapy for a chronic pain population.

## Introduction

Chronic pain represents a major health problem internationally. For example, approximately 30–50% of individuals will suffer clinically significant neck-pain annually (Hogg-Johnson et al., 2008; Fernández-de-las-Peñas et al., 2011), and 50% of the United Kingdom population will suffer, at least once, an instance of shoulder pain (Hand et al., 2008). In addition to this, it is suggested that most people will suffer low back pain at some point in their lives (Fernández-de-las-Peñas et al., 2011; Cohen, 2015; Carnes et al., 2017).

The problem of chronic pain becomes more problematic when considering that psychological suffering often co-occurs (possibly bi-directionally) with pain. For example, depression, anxiety, and social isolation are reported in 35% of chronic pain cases (Frewen et al., 2008; Gillanders et al., 2014). One explanation as to why this relationship exists arrives in form of *psychological inflexibility.* This can be defined as being excessively entangled in experiential avoidance as a result being cognitively fused, lacking acceptance and openness to painful thoughts and feelings, disconnected from the present moment, and without commitment to living in accordance with personal values (Ciarrochi et al., 2010). When psychologically inflexible, the individual may attempt to avoid situations and thoughts that are deemed painful at the expense of valued living. This individual may also adopt rigid behavior patterns which are not aligned to values. *Psychological flexibility* (the opposite of psychological inflexibility) on the other hand is about being open, accepting, aware of thoughts and feelings which unfold in the present moment, and changing or persisting in behavior which are aligned to central goals and values (Kashdan and Rottenberg, 2010).

Psychological flexibility is specifically a key component of acceptance and commitment therapy (ACT), where the ultimate goal of ACT is to improve mental functioning through increasing psychological flexibility (Hayes et al., 1999). ACT differs from second wave therapies such as cognitive behavioral therapy (CBT) (Beck, 2011) as CBT focuses on symptom reduction to improve mental functioning, whereas, ACT focuses on acceptance, connecting with the here and now (mindfulness), cognitive defusion, as well as identifying and committing to values in order to increase psychological flexibility. ACT is just one of several third wave CBT models, which include; dialectical behavioral therapy (DBT) (Linehan, 2018), metacognitive therapy (MCT) (Wells, 2002) and Mindfulness-based Cognitive Therapy (MBCT) (Segal et al., 2018). Though there are commonalities that overlap between some of these and ACT such as mindfulness and acceptance, the measures used in this present study such as psychological flexibility and cognitive fusion come out of ACT literature specifically, so the focus here is primarily related to ACT implications.

The role of psychological flexibility has been identified in many areas of mental health (Bond and Bunce, 2000; Hayes et al., 2004, 2006; Gaudiano and Herbert, 2006), and also in the area of chronic pain, with most studies suggesting that high levels of psychological flexibility are associated with better functioning outcomes for those in chronic pain (McCracken et al., 2004, 2007, 2014; McCracken and Yang, 2006; McCracken and Samuel, 2007). A recent meta-analysis identified 25 RCTs which explored the efficacy of acceptance and mindfulness-based studies (Veehof et al., 2016). For example, one study has found that after a guided internet-delivered ACT intervention for chronic pain patients, the degree to which individuals engaged in activities increased and pain-related distress, anxiety, and depressive symptoms reduced (Buhrman et al., 2013). In another study, exposure and acceptance strategies were found to reduce fear of movement and depression, whilst increasing overall life satisfaction in people with chronic pain (Wicksell et al., 2008).

In addition to these efficacy-based studies, psychological flexibility has also been explored in terms of associations with physical and psychological functioning. For example, psychological flexibility was found to mediate the effect of online ACT for a chronic pain population in relation to outcomes of anxiety, depression, pain, and mental and physical health (Lin et al., 2018). In addition to this, baseline psychological inflexibility was found to moderate outcome pain interference (the impact of pain on the individual’s life) (Probst et al., 2019). Indeed, most of the studies conducted exploring associations of psychological flexibility and functioning come from McCracken and colleagues. For example, in one study McCracken and Yang (2006) used hierarchical regression to demonstrate that successfully living in accordance with personal values was negatively associated with disability, depression, and pain related anxiety.

McCracken and colleagues, like this present study, has focused on several measures from the ACT literature such as success (or discrepancy) at living in accordance to values, mindfulness, and cognitive fusion. Success at living in accordance to values in ACT refer to how successful an individual is at living in a way which is deemed as important to the individual, such as ‘being healthy,’ ‘being kind,’ ‘being educated,’ ‘developing personal growth.’ Alternatively, the measure of values-discrepancy can be defined as the degree to which the individual is unsuccessful at living in accordance with important values (McCracken and Yang, 2006). In terms of mindfulness, Kabat-Zinn (2003) defines mindfulness as paying attention to the present moment, and non-judgmentally. Connecting with the present moment mindfully prevents the individual from focusing too much on the future or the past, where they may excessively experience painful thoughts and memories. Cognitive fusion can be defined as the tendency of behavior to be overly regulated by cognition, where, for example, a person acts on thoughts as though they are literally true. Whilst cognitive defusion (the opposite of cognitive fusion) is the distancing of thoughts where the person does not act as though these thoughts were literally true (Gillanders et al., 2014).

In another example of using some of these measures, the association of mindfulness with mental health outcomes were explored (McCracken et al., 2007). It was found that after controlling for patient background variables such as pain intensity and pain related acceptance, higher measures of mindfulness were associated with reduced depression, pain related-anxiety, and physical disability (i.e., a negative associate was found). McCracken and Vowles (2008) demonstrated that psychological flexibility measures such as acceptance of pain and mindfulness accounted for significant variance of depression and anxiety. In another study (McCracken and Velleman, 2010), it was demonstrated that success at living in accordance with values, acceptance of pain, and mindfulness accounted for more of the variance of a health status measure than pain intensity scores.

These studies have provided important evidence about how psychological flexibility measures predict mental, social, and physical functioning. However, what these studies have not investigated in depth is the role of aging in predicting mental health and cognitive fusion, and through exploring mediators of these variables within a chronic pain and heterogeneously aged population. Aging is a very important area with so many changes through one’s life from social to cognitive decline, which involve both biological or cognitive components (Schönknecht et al., 2005). In addition to this, the global population is aging rapidly, where the World Health Organisation have reported that between 2015 and 2050 the proportion of the population over 60 years of ages will have nearly doubled from 12 to 22%. They highlight that mental health and well-being should be regarded as equally important for this age group as any other age group, and that approximately 15% of the population over 60 suffer from a mental health disorder (World Health Organization, 2017). It is also suggested that mental health problems are as common in older adults as they are in younger ones, but is less likely to be volunteered, detected, or treated as older people are less likely to complain about losses (e.g., relationships or abilities) as these may be considered normal (NHS England and NHS Improvement, 2017). So, given this importance, the purpose of the proposed study is to investigate this relation between age, psychological flexibility, and metal health more closely and with the use of mediated analysis to explore any indirect relations and identify any processes.

Age has of course been used as a background variable in many previous studies as a standard demographic. One study, outside of chronic pain, which has made reference to age difference suggested that psychological flexibility had been found to decrease with age, however, this was a longitudinal study exploring aging through childhood to early adolescence and over a 6 year period (Williams et al., 2012). Though longitudinal studies maybe regarded as superior to cross-sectional studies as they can directly capture any transitional change through the individuals’ lives, this period of life maybe different to other periods of life as a higher prevalence of anxiety have been found in adolescence as compared to childhood and adulthood (Roza et al., 2003). So, in this instance, a decrease in psychological flexibility as children mature into adolescence maybe expected.

In addition to this, several of the already mentioned studies by McCracken and colleagues have explored age as a background variable in relation to psychological flexibility and related outcomes. For example, in the McCracken and Yang study (McCracken and Yang, 2006) correlational analysis revealed that values success and discrepancy scores were not significantly associated with age [mean age = 47.6 years (*SD* = 11.7)]. In another study, it was found that mindfulness was unrelated to age [mean age = 46.9 years (*SD* = 12.5)] (McCracken et al., 2007). However, McCracken and Vowels showed that there was a positive correlation between age and change in the activity engagement scale (*r* = 0.20, *p* < 0.05) [mean age = 48.1 years (*SD* = 11.0)] (McCracken and Vowles, 2008). In yet another study, mindfulness, psychological acceptance, and values based action were positively correlated with age, suggesting an increase in psychological flexibility with age (*r* = 0.36, *p* < 0.001; *r* = 0.15, *p* < 0.05; and *r* = 0.25, *p* < 0.001 respectively) [mean age = 61.5 years (*SD* = 13.7)] (McCracken and Velleman, 2010).

Therefore, the last two papers (McCracken and Vowles, 2008; McCracken and Velleman, 2010) seem to be in contrast with other work by McCracken and Yang (2006) and McCracken et al. (2007) in the sense that the former found associations between age, psychological flexibility and functioning whilst the later did not. As age sometimes correlates with psychological flexibility and at other times does not it is perhaps because age and psychological flexibility are indirectly related to a third variable such as mental health. So, again, this highlights the need to explore mediating factors in relation to this.

A lot of work has already been conducted in exploring mediation factors for assessing the process of change as a result of an intervention rather than just the outcome. This has been particularly prevalent in the work of ACT as psychological flexibility has been identified as a mediator for ACT-based interventions involving smoking cessation (Gifford et al., 2004), burnout at work (Hayes et al., 2004), stress management (Bond and Bunce, 2000), diabetes management (Gregg et al., 2007), depression (Hayes et al., 2006), and psychosis (Gaudiano and Herbert, 2006). Mediational analysis is particularly important in ACT because it involves functional processes linked to psychological flexibility through six core properties; acceptance, cognitive defusion, focusing on the present moment, enhancing a transcendent sense of self, values clarification and committed action, which can be identified indirectly through mediation analysis rather than just outcome measures (Ciarrochi et al., 2010). Understanding the processes of models like ACT help researchers understand which components of the therapy are actually causing the positive change in the mental health or general functioning of an individual when given an intervention.

In terms of specific hypotheses, as the associations between age and measures of psychological flexibly have not always been found to be significant the hypotheses here are largely exploratory. As some studies have found that age is related to psychological flexibility (McCracken and Vowles, 2008; Williams et al., 2012), then the first hypothesis to be tested is that age will predict cognitive fusion (a measure of psychological inflexibility), and independent of other predictors (in a hierarchical regression) such as values-discrepancy, mindfulness, and pain intensity. Secondly, as there is evidence that psychological flexibility predicts mental health (Bond et al., 2008; McCracken and Vowles, 2008), and as has been found to mediate improved mental health after ACT-based interventions (Bond and Bunce, 2000; Hayes et al., 2004, 2006; Gaudiano and Herbert, 2006) then the second hypothesis is that age will not significantly account for the variance of mental health when psychological flexibility measures are added in a hierarchical regression (i.e., a null hypothesis is predicted), as the psychological flexibility measures will account for this variance. Thirdly, as psychological flexibility has been found to mediate improved mental health after ACT-based interventions (Bond and Bunce, 2000; Hayes et al., 2004, 2006; Gaudiano and Herbert, 2006), then the third hypothesis is that any relation between age and mental health will be mediated by the degree to which people are psychologically flexible. Conversely, the fourth hypothesis is that it is assumed that mental health will not mediate the relation between age and cognitive fusion as psychological inflexibility (cognitive fusion) will account for most of the variance.

## Materials and Methods

### Participants

The inclusion criteria for this study was that the participants must have been in pain currently and for a continuous period of at least 3 months, over the age of 18, have good ability to read English with normal or corrected to normal vision, and with access to an internet connection (as this study was online). The survey was open to everyone within this inclusion criteria, regardless of gender, ethnic, religious, geographical, and socioeconomical background (education, income, or social class). The exclusion criteria involved anyone who has experienced pain for less than 3 months, under the age of 18, those who did not have an internet connection, and who did not have good comprehension of English with normal or corrected to normal vision. No test, or medical records were examined, inclusion was determined on the basis of the participant’s own accounting and consent.

Data sets were obtained from 233 adult participants (62 participants either left the study without completing the study or were excluded due to not fitting the inclusion criteria – only completed datasets were analyzed), of whom 176 were female, and 57 were male (see Table 2). The age range for participants was 20 to 85 years of age and the mean age overall was 53.12. Participants were also asked to indicate where in the body they felt their pain. The human body was divided into 14 commonly known regions. Multiple areas could be named, and an ‘other’ category was also available. The lower back was the most commonly named painful area (58.3%), followed by the thigh area (47.2%), the shoulders (43.1%) and neck (38.6%) (in most cases participants had pain in multiple areas).

### Materials

#### Survey Monkey^1^

This study utilized the Survey Monkey service to create an online questionnaire. A link was then distributed through the Centre for Innovative Ageing email list at a United Kingdom University, as well as through Facebook, posted on various pain groups.

#### Demographic Questionnaire

The demographic questionnaire asked details about age, gender, bodily areas affected by the pain (with multiple choices given), and the length of time the patient had suffered with this pain.

#### Short Form McGill Pain Questionnaire (SF-MPQ)

This is a scale for rating the quality and intensity of patient pain. It uses 15 descriptors (11 sensory and four affective) which are rated on an intensity scale. This measure has high test–retest reliability (0.45–0.70), as well as validity as it correlates well with the other pain questionnaires such as the Western Ontario and McMasters Universities Pain Scale (*r* = 0.36, *p* < 0.01) (Hawker et al., 2011).

#### General Health Questionnaire 12 (GHQ**)**

This is a psychometric screening tool to identify common psychiatric conditions, and is a widely-utilized measure of mental health (Hu et al., 2007). It assesses the patients’ current mental state in relation to differences to their usual mental state, therefore, it is sensitive to short term psychiatric disorders. The internal consistency of the GHQ-12 is high as it has a Cronbach’s alpha of 0.81 (Politi et al., 1994). The same study also reported a high test–retest reliability, as did another (Quek et al., 2001), who reported a total GHQ-12 intraclass correlation coefficient of 0.79 (*p* < 0.001).

#### Cognitive Fusion 7-Item Questionnaire (CFQ-7)

One of the central processes in ACT to account for psychological inflexibility is cognitive fusion. In the present study, the seven item Cognitive Fusion Questionnaire (CFQ-7) was used as it promises to be the first widely accepted measure of cognitive fusion (Gillanders et al., 2014). The measure has been shown to have good internal consistency and is a valid and valuable measure of pain related cognitive fusion and acceptance (McCracken et al., 2014). The CFQ-7 has shown satisfactory internal consistency and reliability (Cronbach’s α = 0.88–0.92, and test–retest reliability, *r* = 0.71) when tested over a 4-week period (Gillanders et al., 2014).

#### Mindfulness Attention Awareness Scale (MAAS)

This measure has 15 items and is used to measure how aware of moment to moment experiences participants are. Mindful self-awareness can be cultivated using mindfulness training, and the absence of this skill correlates with decreased self-awareness (Brown and Ryan, 2003). The items are rated on a scale from 1 (almost always) to 6 (almost never) and are then averaged. Consistency and validity of the MAAS is high (McCracken et al., 2007), and another study (Frewen et al., 2008) reported a Cronbach’s alpha of 0.83.

#### Chronic Pain Values Inventory (CPVI)

The Chronic Pain Values Inventory (CPVI) is a commonly used measure of values-based action, and is used to find out how successful a person is at living life in accordance with personal, non-material values (McCracken and Yang, 2006). The CPVI assesses the success and the discrepancy between six domains of values (family, intimate relations, friends, work, health, and growth/learning). The measure uses Likert scales from 0 to 5 that indicate the importance of a particular value (0 = *not at all important*; 5 = *extremely important*) and the respondent’s success in living according to that value (0 = *not at all successful;* 5 = *extremely successful*). Scoring yields a mean success rating and a mean discrepancy rating. The measure is based on the presumption that greater suffering results from low success in living in accordance with an important value. Therefore, low congruence between success at living in accordance with an unimportant value has a less negative impact than low congruence at living in accordance with an important value. The importance ratings thus may be used to gauge the weight of the individual success scores (McCracken and Yang, 2006). The CPVI has good internal consistency for the success subscale (Cronbach’s alpha of 0.90) (Foote et al., 2016), however, no test–retest reliability have been identified, to-date (Vowles et al., 2014).

### Procedure

The data was collected from a heterogenous group (i.e., large age range). A dual sampling strategy was used to gather responses, using; (1) Facebook, posting on general pain sites, and (2) Swansea University’s Centre for Innovative Ageing who provided a list of possible contacts within the community of older aged individuals. The survey was open to everyone who fulfilled the inclusion criteria (see section “Participants”).

Once the participants responded to the advertisement by entering the Survey Monkey website they were then presented with an information sheet describing the study in more detail, and a consent sheet. Once they consented they began filling out the demographics section which asked them their age, gender, how long they had consistent pain and where this pain was located (with various options to choose from). They then were presented with the questionnaires (see section “Materials”) to fill out. Participants had to complete all the questions in each section before moving onto the next section, i.e., a programmed rule in Survey Monkey was created preventing participants from proceeding to the next section without completing all of the questions in previous sections first. Participants were allowed to leave the study at any time.

### Ethics Statement

Ethics were approved through Swansea University’s Research Ethics Council (REC), which included obtaining written informed consent from all participants, right to withdraw and a full debriefing at the end of the study in accordance with the Declaration of Helsinki.

### Data Analysis

**FIGURE 1:**
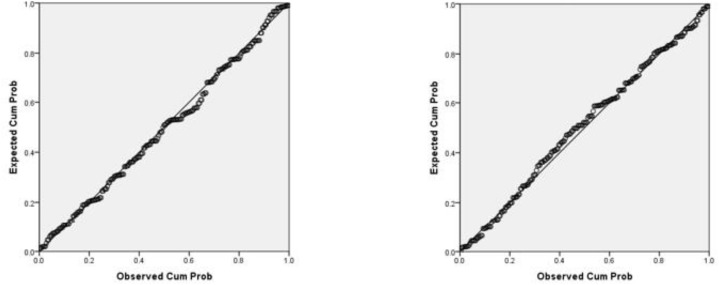
A test of normality using the p–p plot of regression standardized residuals where cognitive fusion is the DV on the left and mental health on the right.

**FIGURE 2:**
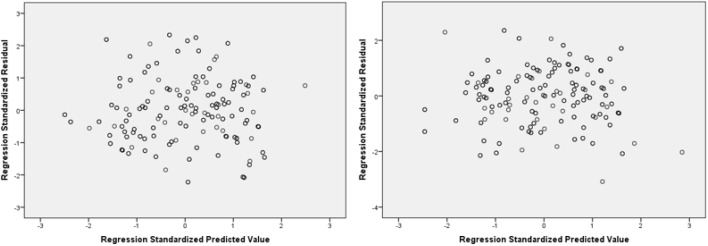
A test for homoscedasticity with a scatterplot of regression standardized residuals with cognitive fusion as a DV on the left and mental health on the right.

Firstly, missing data was averted as the questions for each section needed to be 100% complete before proceeding to the next section of questions (see section “Procedure”). The descriptive and inferential statistics were conducted in SPSS 20.0. Preliminary analyses were first conducted to ensure that there were no violation of the assumptions of normality, linearity, homoscedasticity, and multicollinearity. As you can see in Figure 1, the residuals were linear and normally distributed. Figure 2 shows they were also spread equally so were homoscedastic. All of the correlations between predictors had an *r* value of below 0.8 (see Table 3), and the VIF scores of the coefficients were all below 10 indicating that these were free from multicollinearity.

Hierarchical regressions were used instead of stepwise regressions. In a stepwise regression the computer decides which variables are entered and at what staged based on, for example, a selection criteria. For example, the predictors will be retained if they improve the overall goodness of the fit of the model (*R^2^*) while minimizing the complexity of the model through the Akaike information criterion (AIC); which is founded on information theory (Akaike, 1974). Hierarchical regression on the other hand allows the researcher to choose the variables to enter and at what stage, basing this cumulative procedure according to some pre-specified hierarchy that the researcher has identified through knowledge, purpose, and logic of the research instead of an automated goodness fit.

**FIGURE 3:**
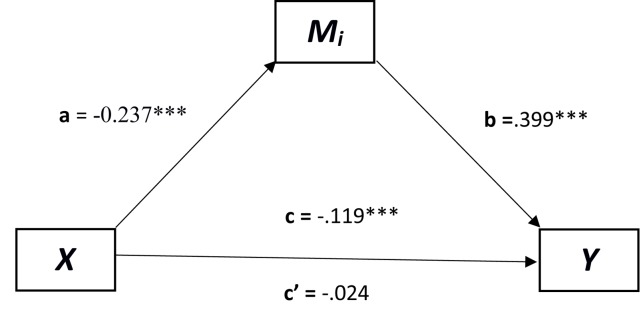
A mediated regression where age predicts mental health and cognitive fusion mediates this relationship. *X* = predictor; *Y* = dependent variable; *Mi* = mediator. ^∗^*p* < 0.05, ^∗∗^*p* < 0.01, ^∗∗∗^*p* < 0.001.

Finally, two mediation analyses were conducted using the SPSS PROCESS (model 7: see Figure 3) macro by Andrew Hayes (Hayes, 2013) with 5000 bootstrap samples and 95% confidence intervals, to explore whether cognitive fusion mediates the relation between age and mental health, and whether mental health mediates the relation between age and cognitive fusion.

## Results

### Indexing and Descriptive Statistics

Table 1 shows the scoring index used for these questionnaires, whilst Table 2 shows the descriptive statistics for the questionnaire scores. As can be seen by the descriptive statistics, pain duration averaged at 7.13 (*SD* = 1.628) months and age was 53.12 (*SD* = 14.81) years with a range between 20 and 85 years of age. In addition to this, the skewness and kurtosis measures are all within plus or minus two except for symptom duration which indicates a normal distribution for each variable (George and Mallery, 2010).

**Table 1:** Index of what do the measure scores mean.

Outcome measure/variable	Meaning
SFMPQ Total Sen + Aff + PPI	Higher scores reflect more intense pain, and more interference with daily life
MAAS	High scores reflect more mindfulness
CPVI Mean Discrepancy (Value Importance – Success)	This is a mean discrepancy rating (importance – success) Higher discrepancy scores represent greater suffering
CPVI Avg. of Success Scores	This is a mean values success rating (avg. of the 6 success ratings). Higher scores represent higher success.
GHQ Total Score	Higher scores represent worse mental health
CFQ-7 Total Score	Higher scores represent higher cognitive fusion

*Sen, sensory; Aff, affective; PPI, present pain intensity.*

**Table 2:** Descriptive statistics and normality scores for the variables.

Variable	M (SD)	Minimum–maximum	Skewness	Kurtosis
(1) Values discrepancy	1.345 (1.053)	-0.67, 4.17	0.457	-0.388
(2) Values success	2.519 (1.011)	0.33, 4.83	-0.214	-0.668
(3) Mindfulness	4.004 (0.948)	2, 6	0.21	-0.893
(4) Pain intensity	16.557 (7.614)	1, 33	-0.010	-0.784
(5) Pain duration	7.13 (1.628)	3, 8	-1.964	3.018
(6) Age	53.12 (14.81)	20, 85	-0.506	-0.665
(7) Mental health	18.939 (6.836)	1.0, 35.0	-0.290	-0.255
(8) Cognitive fusion	25.939 (10.596)	7.0, 49.0	-0.53	-0.797

### Correlations Amongst Variables

Table 3 shows the correlations between the different measures. Background demographics such as gender correlated only with values discrepancy, whilst symptom duration positively correlated only with pain intensity. Age positively correlated with mindfulness, values success, and negatively with mental health and cognitive fusion.

**Table 3:** Correlations.

	1	2	3	4	5	6	7	8
2	0.088							
3	-0.124	0.063						
4	0.128	0.205^**^	-0.098					
5	-0.075	-0.097	0.371^***^	-0.276^***^				
6	0.201^**^	-0.007	-0.121	0.428^***^	-0.131^*^			
7	-0.119	-0.097	0.130^*^	-0.480^***^	0.209^**^	-0.739^***^		
8	0.020	0.089	-0.259^***^	0.496^***^	-0.533^***^	0.412^***^	-0.552^***^	
9	0.107	0.101	-0.332^***^	0.377^***^	-0.559^***^	0.304^***^	-0.366^***^	0.637^***^

*1 = gender, 2 = symptom duration, 3 = age, 4 = pain intensity; 5 = mindfulness; 6 = values discrepancy; 7 = values success, 8 = mental health; 9 = cognitive fusion. ^∗^p < 0.05, ^∗∗^p < 0.01, ^∗∗∗^p < 0.001.*

### Hierarchical Regressions

Table 4 shows the steps of the first hierarchical regression model. In this model the criterion dependent measure (DV) was mental health. The first predictor (IV) added in step one was values discrepancy, followed by mindfulness in step two, then pain intensity in step three, and then finally age in step four. As can be seen, when controlling for values discrepancy, mindfulness and pain intensity, age did not significantly increase the accounting of variance for mental health.

**Table 4:** Model summary of steps where variables are predicting mental health.

Predictors	Adjusted *R*^2^	Δ*R*^2^	β	CI (95%)	Model *F(df), p*
Step 1					
Values discrepancy	0.166	0.170^***^	0.412^***^	[1.906, 3.439]	47.184 (232), *p* < 0.001
Step 2					
Values discrepancy	0.398	0.233^***^	0.348^***^	[1.603, 2.917]	77.641 (232), *p* < 0.001
Mindfulness			-0.487^***^	[-0.252, -0.157]	
Step 3					
Values discrepancy	0.455	0.059^***^	0.237^***^	[0.856, 2.227]	65.607 (232), *p* < 0.001
Mindfulness			-0.425^***^	[-0.252, -0.157]	
Pain intensity			0.278^***^	[0.104, 0.238]	
Step 4					
Values discrepancy	0.465	0.002	0.233^***^	[0.822, 2.198]	49.468 (232), *p* < 0.001
Mindfulness			-0.406^***^	[-0.246, -0.144]	
Pain intensity			0.280^***^	[0.105, 0.240]	
Age			-00.53	[-0.072, 0.023]	

*^∗∗∗^p < 0.001.*

Table 5 shows the steps of the second hierarchical regression model. In this model the criterion DV was cognitive fusion. The first predictor (IV) added in step one was values discrepancy, followed by mindfulness in step two, then pain intensity in step three, and then finally age in step four. This time, as can be seen, age significantly accounted for the variance in relation to the DV (cognitive fusion) when controlling for values discrepancy, mindfulness, and pain intensity.

**Table 5:** Model summary of steps where variables are predicting cognitive fusion.

Predictors	Adjusted R^2^	Δ*R*^2^	β	CI (95%)	Model *F(df), p*
Step 1					
Values discrepancy	0.089	0.093^***^	0.304^***^	[1.821, 4.305]	23.607 (232), *p* < 0.001
Step 2					
Values discrepancy	0.362	0.274^***^	0.236^***^	[1.320, 3.418]	66.700 (232), *p* < 0.001
Mindfulness			-0.528^***^	[-0.471, -0.316]	
Step 3					
Values discrepancy	0.381	0.022^**^	0.168^**^	[0.556, 2.821]	48.635 (232), *p* < 0.001
Mindfulness			-0.490^***^	[-0.444, -0.286]	
Pain intensity			0.170^**^	[0.051, 0.273]	
Step 4					
Values discrepancy	0.394	0.015^*^	0.156^**^	[0.443, 2.694]	38.636 (232), *p* < 0.001
Mindfulness			-0.442^***^	[-0.413, -0.245]	
Pain intensity			0.175^**^	[0.058, 0.277]	
Age			-0.132^*^	[-0.172, -0.016]	

*^∗^p < 0.05, ^∗∗^p < 0.01, ^∗∗∗^p < 0.001.*

### Mediation

In the first mediation analysis (see Figure 3), cognitive fusion was used as a mediator between age and mental health. The relationship between age and the mediator (*M*_i_) was significant [*a* = *t*(231) = -5.342, *p* < 0.001, *B* = -0.237], as well as the relation between *M*_i_ and mental health [*b* = *t*(230) = 11.518, *p* < 0.0001, *B* = 0.399]. The direct relation between age and mental health before the mediator was added was significant [*c* = *t*(230) = -4.080, *p* < 0.0001, *B* = -0.119]. In addition to this, the relationship between age and mental health once the mediator was added became non-significant [*c*′ = *t*(230) = -1.004, *p* = 0.316, *B* = -0.024]. As *c* to *c′* decreased below significance this indicates a strong mediation effect of cognitive fusion. Finally, the confidence intervals of the indirect effect of *X* on *Y* did not cross 0 [CI = -0.138, -0.058] confirming that cognitive fusion does mediate the relationship between age and mental health.

In the second mediation analysis it was important to test for mental health as a mediator between the relationship of age and cognitive fusion. The relationship between age and the mediator (*M*_i_) was significant [*a* = *t*(231) = -4.4116, *p* < 0.001, *B* = -0.119] as well as the relation between the *M*_i_ and cognitive fusion [*b* = *t*(230) = 11.518, *p* < 0.0001, *B* = 0.915]. The direct relation between age and cognitive fusion before the mediator was added was significant [*c* = *t*(230) = -5.343, *p* < 0.0001, *B* = -0.2373]. However, the relationship between age and cognitive fusion was still significant once the mediator was added though reduced [*c*′ = *t*(230) = -3.478, *p* < 0.001, *B* = -0.127] suggesting that mental health only partially mediated the relation between age and cognitive fusion. Finally, the confidence intervals of the indirect effect of *X* on *Y* did not cross 0 [CI = -0.169, -0.057] confirming that mental health does partially mediate the relationship between age and cognitive fusion.

## Discussion

This paper sought to explore, firstly in a hierarchical regression model whether age predicted cognitive fusion (a measure of psychological inflexibility) independently of other predictors such as values-discrepancy, mindfulness, and pain intensity (it was hypothesized that it would). It also sought to explore if, in a second hierarchical regression, whether age predicted mental health independently of other predictors such as values-discrepancy, mindfulness, and pain intensity (it was hypothesized that it would not, i.e., that a null hypothesis would be found). Thirdly, cognitive fusion was explored as a mediator for the relation between age and mental health (it was hypothesized that it would mediate this relation). Finally, whether, conversely, mental health mediated the relation between age and cognitive fusion (it was hypothesized that it would not, i.e., that a null hypothesis would be found).

The findings revealed that age predicted cognitive fusion independently of predictors; values-discrepancy, mindfulness, and pain intensity in the first hierarchal regression (the first hypothesis was thus found to be true). However, age did not predict mental health independently of predictors; values-discrepancy, mindfulness, and pain intensity in the second hierarchal regression (the second hypothesis (null hypothesis) was found to be true). Cognitive fusion was found to mediate the relation between age and mental health (the third hypothesis was found to be true), but mental health was not found to be a good mediator for the relation between age and cognitive fusion (the fourth hypothesis (null hypothesis) was found to be true), as it only partially mediated the relation.

These findings are very interesting, as firstly, the second hierarchical regression demonstrates that age does not account for the variance in mental health (whereas it did account for cognitive fusion in the first hierarchical regression), and instead the variance is accounted for by the measures relating to psychological flexibility and pain intensity. Further to this, it was interesting to find that cognitive fusion mediated the relation between age and mental health which further verified that psychological flexibility accounted for the variance between age and mental health. This indicates that psychological flexibility is an important construct to explore when measuring change in mental health as people age and provides some evidence of the process (psychological flexibility) between age and mental health when aging.

These findings add to the growing body of literature which demonstrates that psychological flexibility mediates mental health and other functioning outcome measures (Bond and Bunce, 2000; Hayes et al., 2004, 2006; Gaudiano and Herbert, 2006). This also includes the area of chronic pain, where it was found that psychological flexibility mediated pre and post pain related disability and life satisfaction for patients with chronic pain following whiplash who underwent exposure-based behavioral cognitive treatment (Wicksell et al., 2010). These results offer some new insights into the relationship between aging and mental health and in the context of chronic pain which have not been previously explored. This work supplements that of McCracken and colleagues. McCracken and colleagues had explored primarily psychological flexibility as predictors for psychological functioning (McCracken and Yang, 2006; McCracken et al., 2007; McCracken and Vowles, 2008; McCracken and Velleman, 2010; McCracken and Zhao-O’Brien, 2010) whereas this present study explored specifically how aging and mental health are related and indirectly related by a psychological flexibility measure (cognitive fusion).

Investigating indirect relations in the form of processes are important. In the last few decades research has focused primarily on the efficacy of packages of interventions such as CBT, dialectic behavior therapy, stress inoculation therapy, behavioral martial therapy, interpersonal therapy, ACT, and many others for a range of disorders (Chambless and Ollendick, 2001). Whilst these package approaches, where the focus is on the outcomes are useful for identifying efficacy and acceptability, they do have limitations. For example, simply focusing on outcomes does not tell the researcher or the clinician which aspects of the intervention are particularly useful, and it does not prevent researchers and clinicians from endlessly re-packaging existing therapies. For this reason, it is important to explore the processes through mediation analysis of the intervention as well as the underlaying functioning of the mental health disorders being considered (Rosen and Davison, 2003). This is similarly important when exploring the process of a relation such as age and mental health. By identifying that psychological flexibility (cognitive fusion) mediates the relation between age and mental health, more effort can be placed on utilizing interventions which promote psychological flexibility, such as ACT, for an aging population with growing mental health disorders. This maybe particularly useful from a clinical health policy perspective as it may help clinicians avoid the use and development of other interventions which may not promote psychological flexibility as well and hence may be les effective and ultimately more costly.

In addition to this, though much of the focus here has been based on ACT (as concepts and measures such as psychological flexibility and cognitive fusion come out of the ACT literature) these findings may generalize onto other third wave therapies which incorporate concepts similar to ACT such as acceptance and mindfulness, for example; DBT (Linehan, 2018), MCT (Wells, 2002) and MBCT (Segal et al., 2018). Practitioners of these therapies do not explicitly suggest that they lead to greater psychological flexibility, but they are likely to. This has already been found in some situations, where, for example, psychological flexibility has been found to mediate MBCT exposure and a reduction in depressive symptoms (Pots et al., 2014) which confirms that increased psychological flexibility is at least one process by which MBCT reduces depression. This, again, is why exploring the process of the therapies are so important, i.e., to understand the common psychological construct that is being promoted in leading to positive metal health outcomes. This construct ‘psychological flexibility’ may also need to be further explored outside of the ACT literature, from the perspective of clinicians and researchers working in the area of cognition, for example, such as DCT and MBCT.

This study of course has limitations. One of the main limitations of this study is that it did not incorporate any measures to ensure the responses were reliable. Work by Oppenheimer et al. (2009) suggest that this is important to add an instructional manipulation check in order check that the participants are actually reading and understanding what is being asked. This could involve, for example, the participants being instructed to press a particular button rather than answer the question in the larger print. As participants tend to reduce the amount of information they have to read, it is suggested by Oppenheimer and colleagues that the less motivated participants will ignore the instructions and just answer the question in the larger print when they should not. Through this process the less motivated participants who are not paying attention to the instructions can be identified and removed from the study, and this will reduce the noise and increase the statistical power according to the study.

Another possible limitation was that a rule was created which forces participants to complete all questions before continuing to the next question set. This was included so as to prevent incomplete questionnaires thus missing data; however, it is recognized that by forcing participants to answer all questions can lead to a greater number of mistakes and incorrect answers (i.e., thus increasing statistical noise) as participants perhaps become frustrated. A third possible limitation was that for the inclusion criteria, participant’s own reporting on whether they had chronic pain for a period of 3 months was used and not actual medical records. Medical records may be a more accurate way of identifying the length of time in pain.

In summary, on the whole, this study has produced some interesting findings in relation to the associations and process between age, mental health, pain and the psychological flexibility measures of cognitive fusion, values discrepancy, and mindfulness. The process models described here are important as they offer insights into the components of the theory which explain them, and these findings offer support to the ACT model as they are consistent with its principles in relation to psychological flexibility and mental health. This work is exciting and further work should now explore these relations in other areas of clinical domains which explore age more closely.

## Data Availability

The datasets generated for this study are available on request to the corresponding author.

## Author Contributions

DE designed, wrote, analyzed, and conducted all of the statistical analysis for this paper.

## Conflict of Interest Statement

The author declares that the research was conducted in the absence of any commercial or financial relationships that could be construed as a potential conflict of interest.

## Footnotes

1 Survey Monkey: https://www.surveymonkey.co.uk
